# Methyl 5-iodo-2-meth­oxy­benzoate

**DOI:** 10.1107/S1600536814005868

**Published:** 2014-03-22

**Authors:** Fredrik Lundvall, David Stephen Wragg, Pascal D. C. Dietzel, Helmer Fjellvåg

**Affiliations:** a Centre for Materials Science and Nanotechnology, Department of Chemistry, University of Oslo, PO Box 1126, 0315 Oslo, Norway; b Centre for Materials Science and Nanotechnology, University of Oslo, PO Box 1033, 0315 Oslo; c Department of Chemistry, University of Bergen, PO Box 7803, 5020 Bergen, Norway

## Abstract

In the title compound, C_9_H_9_IO_3_, the mol­ecules are close to planar [maximum deviation from benzene ring plane = 0.229 (5) Å for the methyl carboxylate C atom] with the methyl groups oriented away from each other. In the crystal, mol­ecules form stacked layers parallel to the *ab* plane, where every layer has either the iodine or meth­oxy/methyl carboxyl­ate substituents pointing towards each other in an alternating fashion.

## Related literature   

For the synthesis, see Wang *et al.* (2009 ▶).
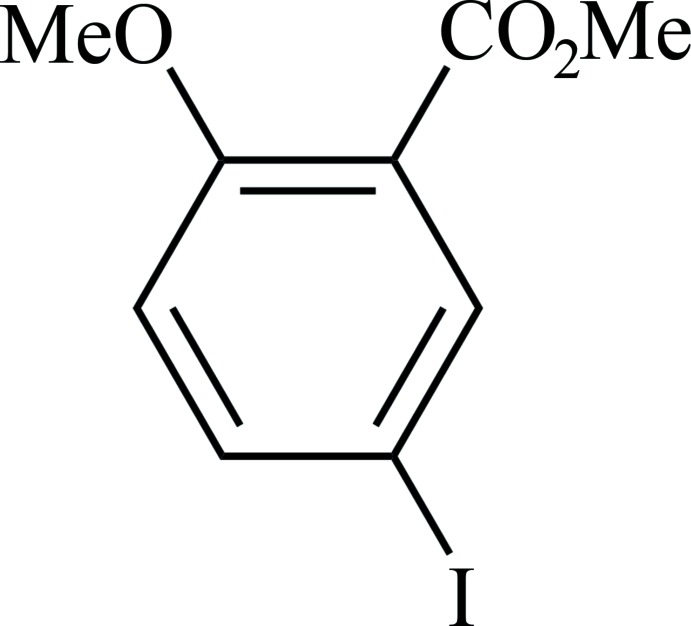


## Experimental   

### 

#### Crystal data  

C_9_H_9_IO_3_
*M* *_r_* = 292.06
Monoclinic, 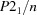

*a* = 4.3378 (7) Å
*b* = 7.0690 (11) Å
*c* = 33.120 (5) Å
β = 92.727 (2)°
*V* = 1014.4 (3) Å^3^
*Z* = 4
Mo *K*α radiation
μ = 3.13 mm^−1^
*T* = 293 K
0.35 × 0.20 × 0.08 mm

#### Data collection  

Bruker APEXII CCD diffractometer
Absorption correction: multi-scan (*SADABS*; Sheldrick, 1996 ▶) *T*_min_ = 0.407, *T*_max_ = 0.788
7578 measured reflections
2064 independent reflections
1971 reflections with *I* > 2σ(*I*)
*R*_int_ = 0.018

#### Refinement  

*R*[*F* ^2^ > 2σ(*F* ^2^)] = 0.033
*wR*(*F* ^2^) = 0.079
*S* = 1.17
2064 reflections
120 parameters
H-atom parameters constrained
Δρ_max_ = 0.67 e Å^−3^
Δρ_min_ = −0.86 e Å^−3^

### 

Data collection: *APEX2* (Bruker, 2007 ▶); cell refinement: *SAINT* (Bruker, 2007 ▶); data reduction: *SAINT*; program(s) used to solve structure: *SIR92* (Altomare *et al.*, 1994 ▶); program(s) used to refine structure: *SHELXL97* (Sheldrick, 2008 ▶) implemented in *WinGX* (Farrugia, 2012 ▶); molecular graphics: *DIAMOND* (Brandenburg, 2004 ▶) and *ChemBioDraw Ultra* (CambridgeSoft, 2009 ▶); software used to prepare material for publication: *publCIF* (Westrip, 2010 ▶).

## Supplementary Material

Crystal structure: contains datablock(s) I, New_Global_Publ_Block. DOI: 10.1107/S1600536814005868/lr2123sup1.cif

Structure factors: contains datablock(s) I. DOI: 10.1107/S1600536814005868/lr2123Isup2.hkl

Supporting information file. DOI: 10.1107/S1600536814005868/lr2123Isup3.cml

CCDC reference: 992130

Additional supporting information:  crystallographic information; 3D view; checkCIF report

## Figures and Tables

**Fig. 1.**
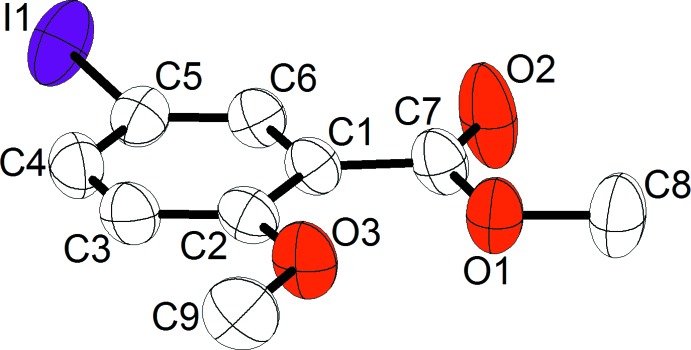
One molecular unit of the title compound with 50% probability displacement ellipsoids. Hydrogen atoms are omitted for clarity.

**Fig. 2.**
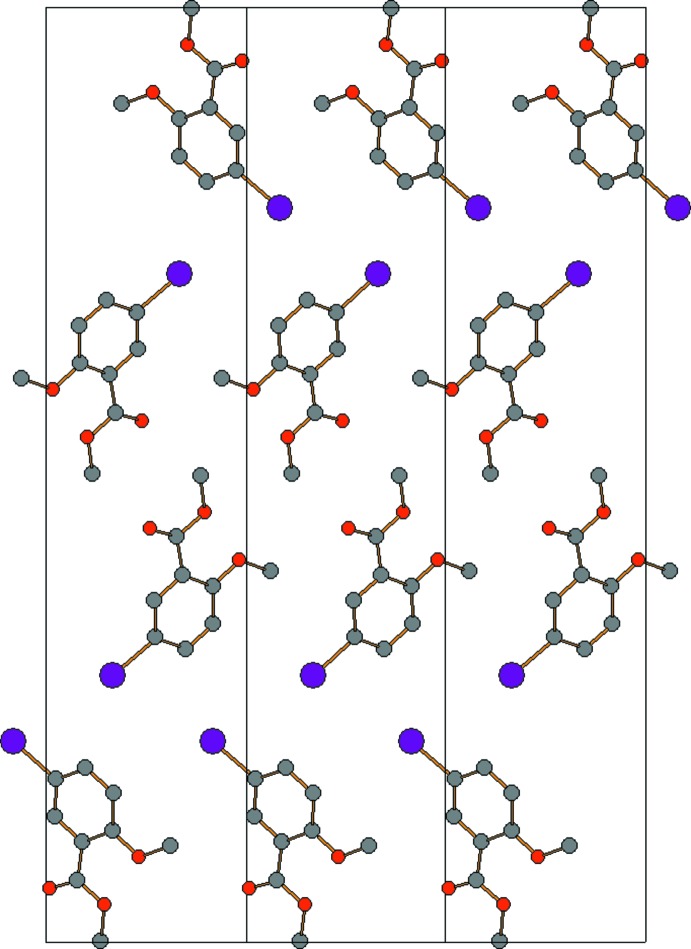
Packing diagram of the title compound viewed along the a axis. Hydrogen atoms are omitted for clarity.

**Fig. 3.**
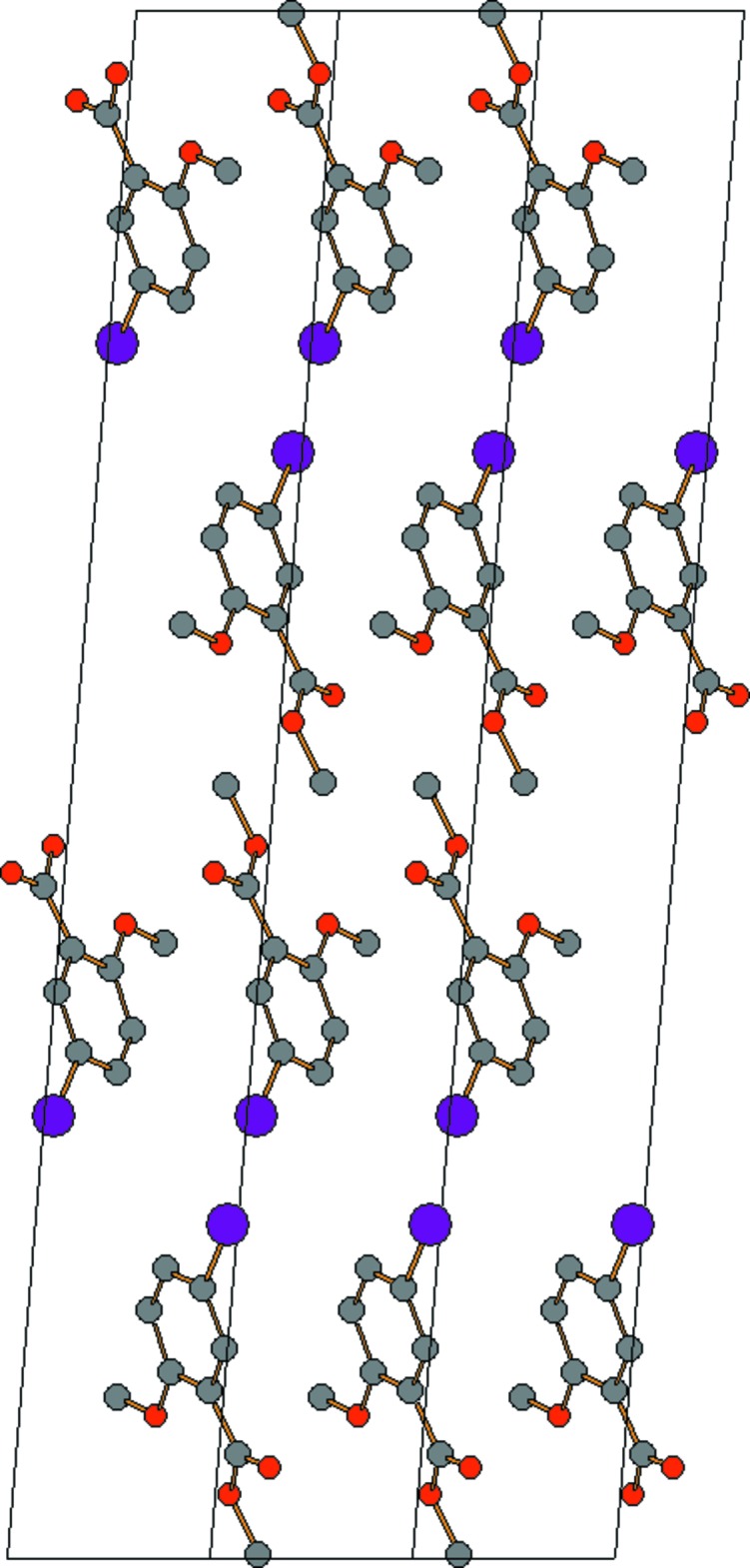
Packing diagram of the title compound viewed along the b axis. Hydrogen atoms are omitted for clarity.

## supplementary crystallographic information

### 2.1. Synthesis and crystallization

The title compound was synthesized by the method used by Wang *et al.* (2009), only differing slightly in the reaction time which was increased from 30 to 60 minutes. The ^1^H NMR spectrum of the title compound is in good agreement with what was reported by Wang *et al.* (2009). The title compound was dissolved in CDCl_3_ for NMR-analysis, and slow evaporation of the solvent yielded single crystals suitable for X-ray diffraction.

### 2.2. Refinement

Crystal data, data collection and structure refinement details are summarized in Table 1. The structure was refined by full-matrix least squares using *SHELXL97* (Sheldrick, 2008) as implemented in the *WinGX* suite (Farrugia, 2012). H-atoms were positioned geometrically at distances of 0.93 (CH) and 0.96 Å (CH_3_) and refined using a riding model with *U*_iso_ (H)=1.2 *U*_eq_ (CH) and *U*_iso_ (H)=1.5 *U*_eq_ (CH_3_).

### 3. Results and discussion

The title compound is an inter­mediate in the synthesis of 4,4'-di­meth­oxy-3,3'-bi­phenyldi­carb­oxy­lic acid, a novel organic linker for use in MOFs (Metal-Organic Frameworks). The title compound is a known inter­mediate from the literature (Wang *et al.*, 2009), but the crystal structure has not been reported so far.

The structure of the title compound, C_9_H_9_IO_3_, has a monoclinic *P*2_1_/*c* symmetry. The asymmetric unit equals one molecule of the compound, with the full content of the unit cell generated by symmetry operations. The molecule has a planar motif where the methyl groups are oriented away from each other to accommodate the sterical demands of these groups. To further increase the distance between the methyl groups, an alternative configuration of the molecule could theoretically be achieved by rotating the methyl carboxyl­ate group 180° around the C1–C7 bond. This however appears not to be an energetically favourable configuration in the solid state. The asymmetric units are packed to form layers parallel to the *C* plane, which results in a layered structure where every other layer has either an iodine or a meth­oxy/methyl carboxyl­ate inter­face.

### Crystal data

**Table:**

C_9_H_9_IO_3_	*F*(000) = 560
*M**_r_* = 292.06	*D*_x_ = 1.912 Mg m^−^^3^
Monoclinic, *P*2_1_/*n*	Mo *K*α radiation, λ = 0.71073 Å
Hall symbol: -P 2yn	Cell parameters from 5646 reflections
*a* = 4.3378 (7) Å	θ = 2.5–28.8°
*b* = 7.0690 (11) Å	µ = 3.13 mm^−^^1^
*c* = 33.120 (5) Å	*T* = 293 K
β = 92.727 (2)°	Plate, colourless
*V* = 1014.4 (3) Å^3^	0.35 × 0.20 × 0.08 mm
*Z* = 4	

### Data collection

**Table:**

Bruker APEXII CCD diffractometer	2064 independent reflections
Radiation source: fine-focus sealed tube	1971 reflections with *I* > 2σ(*I*)
Graphite monochromator	*R*_int_ = 0.018
φ and ω scans	θ_max_ = 26.4°, θ_min_ = 2.5°
Absorption correction: multi-scan (*SADABS*; Sheldrick, 1996)	*h* = −5→5
*T*_min_ = 0.407, *T*_max_ = 0.788	*k* = −8→8
7578 measured reflections	*l* = −41→41

### Refinement

**Table:**

Refinement on *F*^2^	Primary atom site location: structure-invariant direct methods
Least-squares matrix: full	Secondary atom site location: difference Fourier map
*R*[*F*^2^ > 2σ(*F*^2^)] = 0.033	Hydrogen site location: inferred from neighbouring sites
*wR*(*F*^2^) = 0.079	H-atom parameters constrained
*S* = 1.17	*w* = 1/[σ^2^(*F*_o_^2^) + (0.0267*P*)^2^ + 1.5075*P*] where *P* = (*F*_o_^2^ + 2*F*_c_^2^)/3
2064 reflections	(Δ/σ)_max_ < 0.001
120 parameters	Δρ_max_ = 0.67 e Å^−^^3^
0 restraints	Δρ_min_ = −0.86 e Å^−^^3^

### Special details

**Table:**

Experimental. The recrystallization was performed in deuterated solvent, CDCl_3_.
Geometry. All e.s.d.'s (except the e.s.d. in the dihedral angle between two l.s. planes) are estimated using the full covariance matrix. The cell e.s.d.'s are taken into account individually in the estimation of e.s.d.'s in distances, angles and torsion angles; correlations between e.s.d.'s in cell parameters are only used when they are defined by crystal symmetry. An approximate (isotropic) treatment of cell e.s.d.'s is used for estimating e.s.d.'s involving l.s. planes.
Refinement. Refinement of *F*^2^ against ALL reflections. The weighted *R*-factor *wR* and goodness of fit *S* are based on *F*^2^, conventional *R*-factors *R* are based on *F*, with *F* set to zero for negative *F*^2^. The threshold expression of *F*^2^ > σ(*F*^2^) is used only for calculating *R*-factors(gt) *etc*. and is not relevant to the choice of reflections for refinement. *R*-factors based on *F*^2^ are statistically about twice as large as those based on *F*, and *R*- factors based on ALL data will be even larger.

### Fractional atomic coordinates and isotropic or equivalent isotropic displacement parameters (Å^2^)

**Table:**

	*x*	*y*	*z*	*U*_iso_*/*U*_eq_	
C8	1.2399 (13)	0.2713 (7)	0.00134 (13)	0.0750 (13)	
H8C	1.1673	0.1559	−0.0110	0.112*	
H8B	1.4608	0.2677	0.0048	0.112*	
H8A	1.1796	0.3764	−0.0156	0.112*	
C7	1.1645 (9)	0.1539 (5)	0.06620 (11)	0.0513 (8)	
C1	1.0379 (8)	0.1809 (5)	0.10720 (10)	0.0432 (7)	
C2	0.8498 (8)	0.3314 (5)	0.11933 (11)	0.0467 (8)	
C3	0.7587 (9)	0.3356 (5)	0.15924 (12)	0.0541 (9)	
H3	0.6357	0.4345	0.1676	0.065*	
C4	0.8469 (9)	0.1966 (6)	0.18637 (12)	0.0563 (9)	
H4	0.7844	0.2022	0.2128	0.068*	
C9	0.5840 (11)	0.6212 (6)	0.10360 (16)	0.0719 (13)	
H9C	0.5652	0.7115	0.0820	0.108*	
H9B	0.6821	0.6798	0.1270	0.108*	
H9A	0.3825	0.5775	0.1100	0.108*	
C5	1.0295 (8)	0.0478 (5)	0.17421 (11)	0.0486 (8)	
C6	1.1226 (8)	0.0425 (5)	0.13512 (10)	0.0450 (7)	
H6	1.2462	−0.0571	0.1272	0.054*	
O1	1.1074 (7)	0.2925 (4)	0.04032 (8)	0.0614 (7)	
O3	0.7663 (7)	0.4639 (4)	0.09139 (9)	0.0652 (8)	
O2	1.3145 (10)	0.0189 (5)	0.05808 (9)	0.0984 (14)	
I1	1.16959 (7)	−0.16619 (5)	0.215055 (9)	0.07304 (14)	

### Atomic displacement parameters (Å^2^)

**Table:**

	*U*^11^	*U*^22^	*U*^33^	*U*^12^	*U*^13^	*U*^23^
C8	0.101 (4)	0.073 (3)	0.053 (2)	0.023 (3)	0.022 (2)	0.014 (2)
C7	0.065 (2)	0.0454 (18)	0.0436 (18)	0.0166 (17)	0.0017 (16)	−0.0022 (15)
C1	0.0462 (17)	0.0391 (16)	0.0441 (17)	0.0076 (14)	0.0004 (14)	−0.0047 (13)
C2	0.0466 (18)	0.0389 (17)	0.055 (2)	0.0056 (14)	0.0007 (15)	−0.0076 (15)
C3	0.050 (2)	0.049 (2)	0.064 (2)	0.0077 (16)	0.0127 (17)	−0.0122 (17)
C4	0.054 (2)	0.067 (2)	0.049 (2)	0.0002 (18)	0.0088 (16)	−0.0088 (18)
C9	0.075 (3)	0.043 (2)	0.098 (3)	0.026 (2)	0.016 (2)	−0.002 (2)
C5	0.0412 (17)	0.055 (2)	0.0495 (19)	−0.0002 (15)	0.0004 (14)	0.0047 (16)
C6	0.0439 (17)	0.0421 (17)	0.0488 (18)	0.0071 (14)	0.0010 (14)	−0.0018 (14)
O1	0.080 (2)	0.0538 (15)	0.0515 (15)	0.0239 (14)	0.0157 (13)	0.0088 (12)
O3	0.0808 (19)	0.0493 (15)	0.0664 (17)	0.0320 (14)	0.0115 (14)	0.0011 (13)
O2	0.166 (4)	0.076 (2)	0.0566 (18)	0.074 (2)	0.032 (2)	0.0091 (16)
I1	0.05978 (19)	0.0988 (3)	0.06086 (19)	0.01134 (15)	0.00665 (13)	0.03178 (15)

### Geometric parameters (Å, º)

**Table:**

C8—O1	1.446 (5)	C3—C4	1.373 (6)
C8—H8C	0.9600	C3—H3	0.9300
C8—H8B	0.9600	C4—C5	1.388 (5)
C8—H8A	0.9600	C4—H4	0.9300
C7—O2	1.193 (4)	C9—O3	1.434 (4)
C7—O1	1.318 (4)	C9—H9C	0.9600
C7—C1	1.501 (5)	C9—H9B	0.9600
C1—C6	1.384 (5)	C9—H9A	0.9600
C1—C2	1.411 (4)	C5—C6	1.375 (5)
C2—O3	1.354 (4)	C5—I1	2.100 (4)
C2—C3	1.398 (5)	C6—H6	0.9300
			
O1—C8—H8C	109.5	C3—C4—C5	119.8 (4)
O1—C8—H8B	109.5	C3—C4—H4	120.1
H8C—C8—H8B	109.5	C5—C4—H4	120.1
O1—C8—H8A	109.5	O3—C9—H9C	109.5
H8C—C8—H8A	109.5	O3—C9—H9B	109.5
H8B—C8—H8A	109.5	H9C—C9—H9B	109.5
O2—C7—O1	122.4 (3)	O3—C9—H9A	109.5
O2—C7—C1	122.2 (3)	H9C—C9—H9A	109.5
O1—C7—C1	115.3 (3)	H9B—C9—H9A	109.5
C6—C1—C2	118.8 (3)	C6—C5—C4	119.4 (3)
C6—C1—C7	114.7 (3)	C6—C5—I1	119.9 (3)
C2—C1—C7	126.5 (3)	C4—C5—I1	120.7 (3)
O3—C2—C3	123.6 (3)	C5—C6—C1	122.0 (3)
O3—C2—C1	117.8 (3)	C5—C6—H6	119.0
C3—C2—C1	118.6 (3)	C1—C6—H6	119.0
C4—C3—C2	121.4 (3)	C7—O1—C8	115.7 (3)
C4—C3—H3	119.3	C2—O3—C9	118.5 (3)
C2—C3—H3	119.3		
			
O2—C7—C1—C6	3.2 (6)	C3—C4—C5—C6	0.7 (6)
O1—C7—C1—C6	−174.6 (3)	C3—C4—C5—I1	179.7 (3)
O2—C7—C1—C2	−177.6 (4)	C4—C5—C6—C1	−0.5 (5)
O1—C7—C1—C2	4.6 (6)	I1—C5—C6—C1	−179.6 (3)
C6—C1—C2—O3	−179.1 (3)	C2—C1—C6—C5	−0.2 (5)
C7—C1—C2—O3	1.7 (6)	C7—C1—C6—C5	179.1 (3)
C6—C1—C2—C3	0.6 (5)	O2—C7—O1—C8	−0.3 (7)
C7—C1—C2—C3	−178.6 (4)	C1—C7—O1—C8	177.4 (4)
O3—C2—C3—C4	179.3 (4)	C3—C2—O3—C9	2.4 (6)
C1—C2—C3—C4	−0.4 (6)	C1—C2—O3—C9	−177.9 (4)
C2—C3—C4—C5	−0.2 (6)		
